# Supplementary material for: Estrogen signaling in arcuate Kiss1 neurons suppresses a sex-dependent female circuit promoting dense strong bones
Source: Nat Commun. 2019 Jan 11;10:163. doi: 10.1038/s41467-018-08046-4 (PMC6329772; doi:10.1038/s41467-018-08046-4)
Supplement: Supplementary file 1 — Supplementary Information [file 41467_2018_8046_MOESM1_ESM.pdf]

Estrogen Signaling in Arcuate *Kiss1* Neurons Suppresses a Sex-Dependent Female Circuit Promoting Dense Strong Bones

Authors: Candice B. Herber†, William C. Krause†, Liping Wang, James R. Bayrer, Alfred Li, Matthew Schmitz, Aaron Fields, Breanna Ford, Zhi Zhang, Michelle S. Reid, Daniel K Nomura, Robert A. Nissenson, Stephanie M. Correa‡, and Holly A. Ingraham

Supplementary Figures and Tables

1. Figures

Supplementary Figure 1. $Esr1^{Nkx2\text{-}1Cre}$ females exhibit persistence of ERα in some brain regions and loss of fertility.

Supplementary Figure 2. Lower movement and markers of BAT in female $Esr1^{Nkx2\text{-}1Cre}$ mice.

Supplementary Figure 3. $Esr1^{Nkx2\text{-}1Cre}$ mice exhibit increased bone and BMP/Interferon signaling.

Supplementary Figure 4. Confirmation of ARC specific ablation of ERα in intact and OVX $ER\alpha KO^{ARC}$ females.

Supplementary Figure 5. Cortical bone area and pituitary serum hormones in $ER\alpha KO^{ARC}$ females.

Supplementary Figure 6. Increased bone mass in $Esr1^{Kiss1\text{-}Cre}$ but not $Esr1^{Pomc\text{-}Cre}$ female mice.

2. Tables

Supplementary Table 1. Gene expression in femoral bone and bone marrow in $Esr1^{fl/fl}$ and $Esr1^{Nkx2\text{-}1Cre}$ females.

Supplementary Table 2. Sequences of primer pairs used for genotyping mouse alleles.

Supplementary Table 3. Primers and probes for BAT and bone markers.

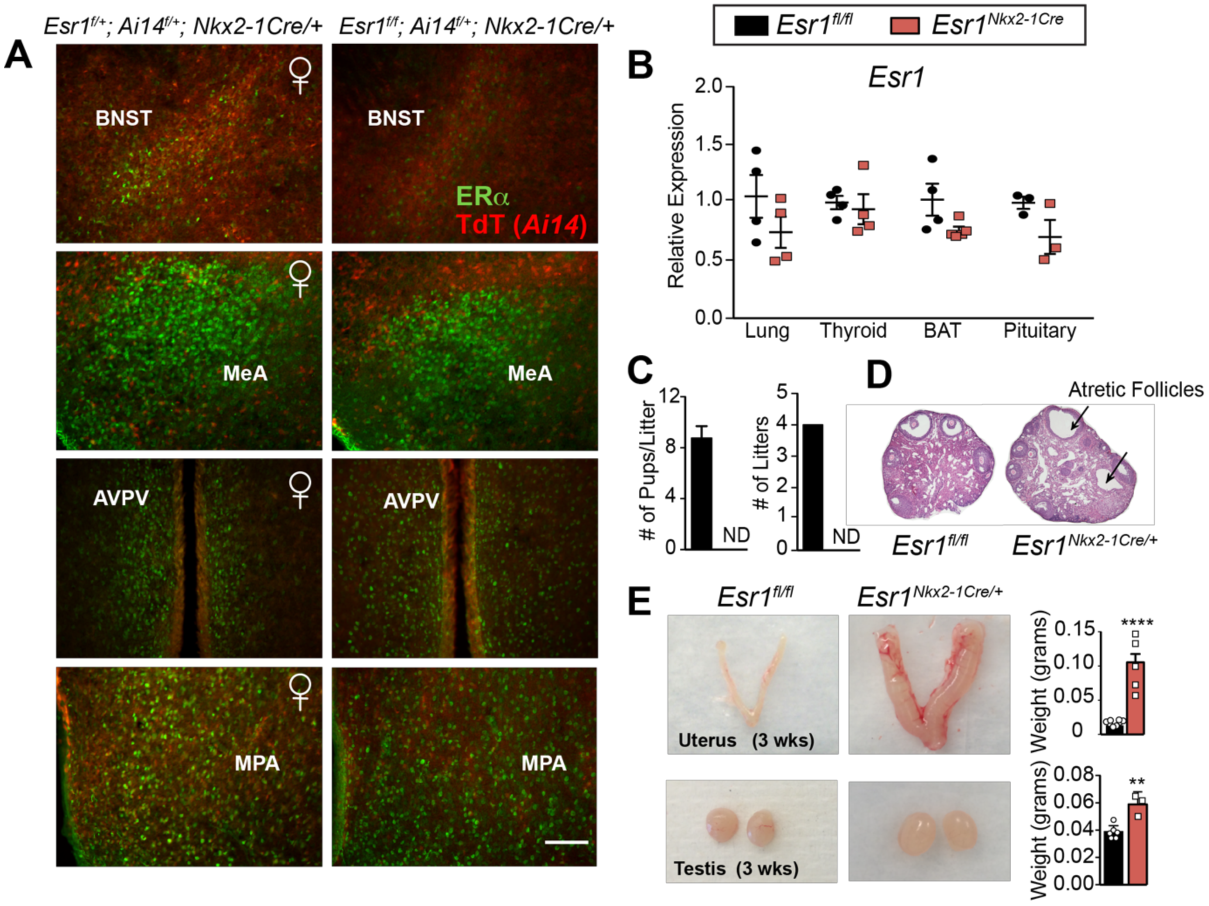

Supplementary Figure 1. ***Esr1****$^{Nkx2\text{-}1Cre}$* **females exhibit persistence of ERα in some brain regions and loss of fertility.**

(**A**) Representative images of ERα immunoreactivity (green) and native TdT expression (red) in coronal brain sections of *Esr1$^{fl/+}$; Ai14$^{fl/+}$; Nkx2-1Cre* and *Esr1$^{fl/fl}$; Ai14$^{fl/+}$; Nkx2-1Cre* females. ERα expression was lost in bed nucleus of the stria terminalis (BNST) but was maintained in the medial amygdala (MeA), anteroventral periventricular nucleus (AVPV) and the medial preoptic area (MPA). Scale bar = 100μm. (**B**) Tissue panel showing no change in *Esr1* transcript levels in *Esr1$^{fl/fl}$* (black circles) compared to *Esr1$^{Nkx2\text{-}1Cre}$* females (red squares). For *Esr1$^{fl/fl}$* females (n = 4) for lung, thyroid and BAT and (n = 3) for pituitary. For *Esr1$^{Nkx2\text{-}1Cre}$* (n = 3) for lung and pituitary and (n = 4) for thyroid and BAT. (**C**) Number of pups per litter and number of litters produced by mating *Esr1$^{fl/fl}$* males and females and *Esr1$^{Nkx2\text{-}1Cre}$* females with

control males. ND = none detected. (**D**) Representative images of ovarian histology showing increased follicular atresia in female mutants (arrows) compared to controls. (**E**) Representative images of uteri and testis of 3 wk old *Esr1*$^{fl/fl}$ and *Esr1*$^{Nkx2\text{-}1Cre}$ mice with right panels showing bar graphs of uterine and testis weight of control (n = 8) and mutant (n =5) females and control (n = 6) and mutant (n =3) males at 3 wks of age. Error bars are ± SEM. Statistical analysis performed was a Student's *t*-test $^{**}p < 0.01$, $^{****}p < 0.0001$.

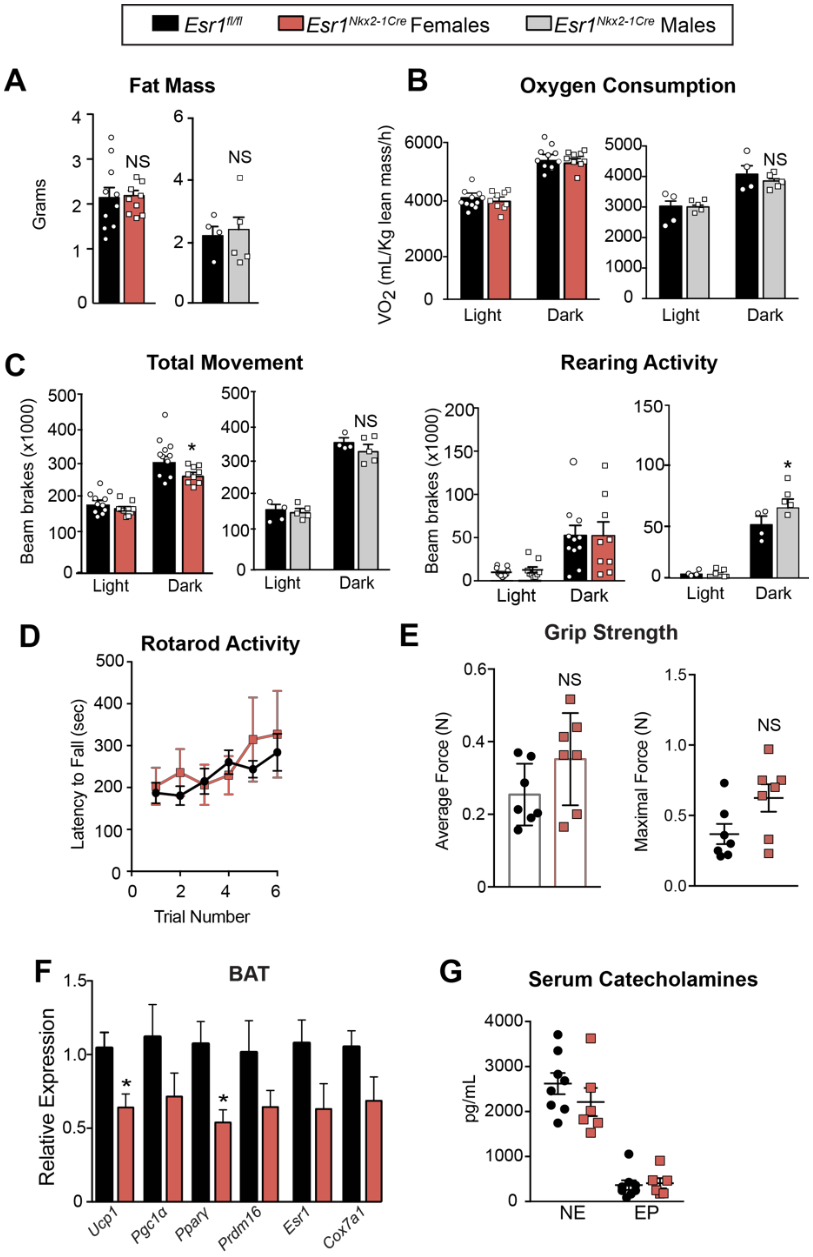

Supplementary Figure 2. Lower movement and markers of BAT in female *Esr1*$^{Nkx2\text{-}1Cre}$ mice.

(**A**) Fat mass determined by EchoMRI. Metabolic chamber analyses of energy expenditure including (**B**) oxygen consumption (VO2) normalized to grams of lean mass, (**C**) and activity as measured by average total movement (beam brakes, x and y axis) ($F_{1,14} = 4.2$, *$p < 0.05$) and cumulative rearing activity (beam brakes, z axis) per animal for 12 h shown for *Esr1*$^{fl/fl}$ (black bars) and *Esr1*$^{Nkx2\text{-}1Cre}$ females (red bars) and males (grey bars, 8-9 wks). (**D**) Rotarod

activity and (**E**) Average and maximal grip strength in control (n = 8, black lines (D) and squares (E)) and mutant (n = 6, red line (D) and red square (E)) females (14 wks) (**F**) Levels of *Ucp1* transcripts and other BAT markers in control and mutant female mice at 22°C (n = 6 controls and n = 8 mutants) at 12-16 wks. (**G**) Serum norepinephrine (NE) and epinephrine (EP) concentrations (7-8 wks). Unless otherwise stated, all experimental groups (n = 11, 9) females and (n = 4, 5) males for controls and mutants, respectively. Error bars are ± SEM. Two-way ANOVA for Panels B and C and Students *t*-test for Panels A, D and E, $^*p < 0.05$, NS = not significant.

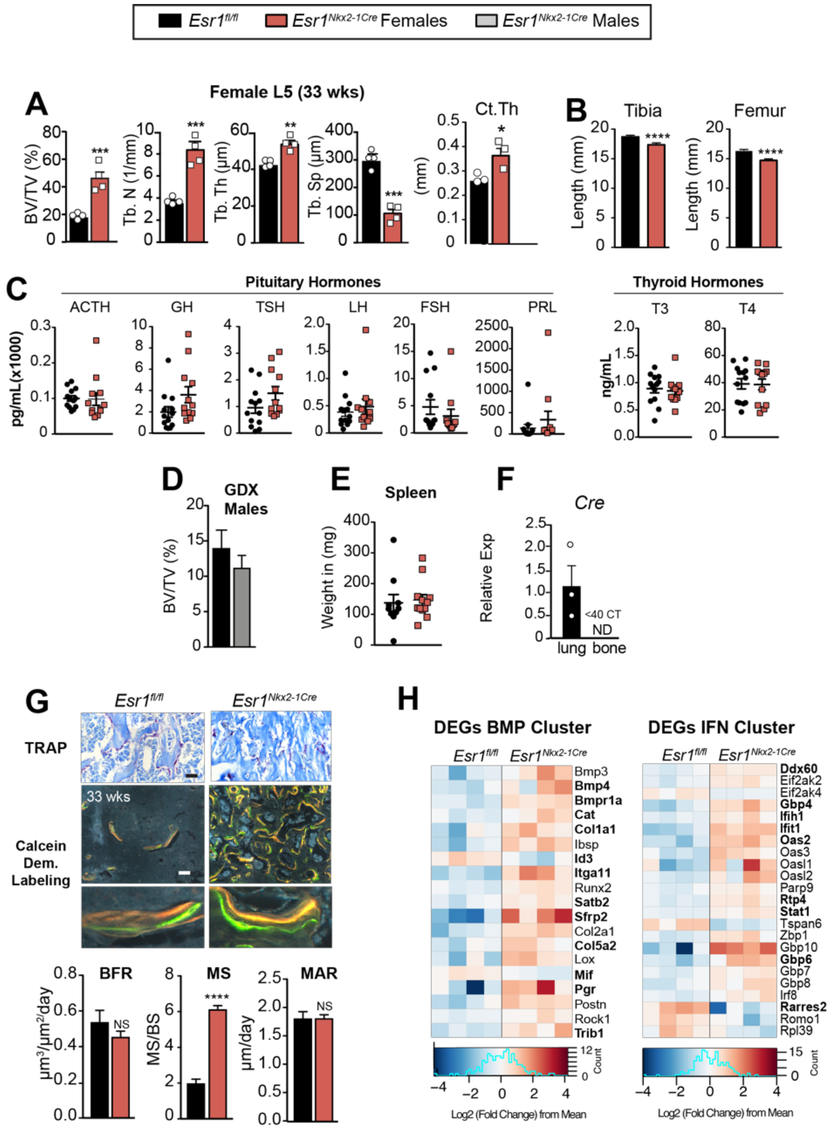

Supplementary Figure 3. $Esr1^{Nkx2\text{-}1Cre}$ mice exhibit increased bone and BMP/interferon signaling.

(**A**) Morphometric properties of L5 vertebrae from 33-wk old $Esr1^{fl/fl}$ (n = 4, black bars) compared to $Esr1^{Nkx2\text{-}1Cre}$ (n = 4, red bars) female mice as analyzed by µCT. Cortical thickness at the TBJ measured by µCT in 20 wk old females in control and mutants (n = 3, 3). (**B**) Morphometric analyses of tibial and femoral length of $Esr1^{fl/fl}$ (n = 5) and $Esr1^{Nkx2\text{-}1Cre}$ (n = 8) female mice (33 wks). (**C**) Plasma concentrations of pituitary and thyroid hormones (7-8 wks). Controls (black squares) and mutants (red squares) (**D**) BV/TV (%) of castrated male femurs (7 wks) (n = 6, black bars) controls and (n = 6, grey bars) mutants. (**E**) Spleen

weights in control and mutant females (7-8 wks). (**F**) *Cre* expression in lung and femoral bone of female mice (n = 3). (**G**) Representative images of TRAP staining in femurs from young females, and from calcein (green) and demeclocycline (orange) labeled femurs (n = 5) controls and (n = 6) mutants with quantified BFR, MS and MAR for older $Esr1^{fl/fl}$ (n = 8) and $Esr1^{Nkx2\text{-}1Cre}$ (n = 5) females (4.5-7 wks). Scale bar = 50μm. (**H**) Heat maps of two significant DEG clusters representing BMP and IFN signaling upregulated in mutant $Esr1^{Nkx2\text{-}1Cre}$ female bone marrow at 4.5 wks of age; scale bars are shown below heap maps. Error bars are ± SEM. Statistical analyses performed using Student's *t*-test, *p < 0.05; **p < 0.01; ***p < 0.001; ****p < 0.0001. NS = p > 0.05.

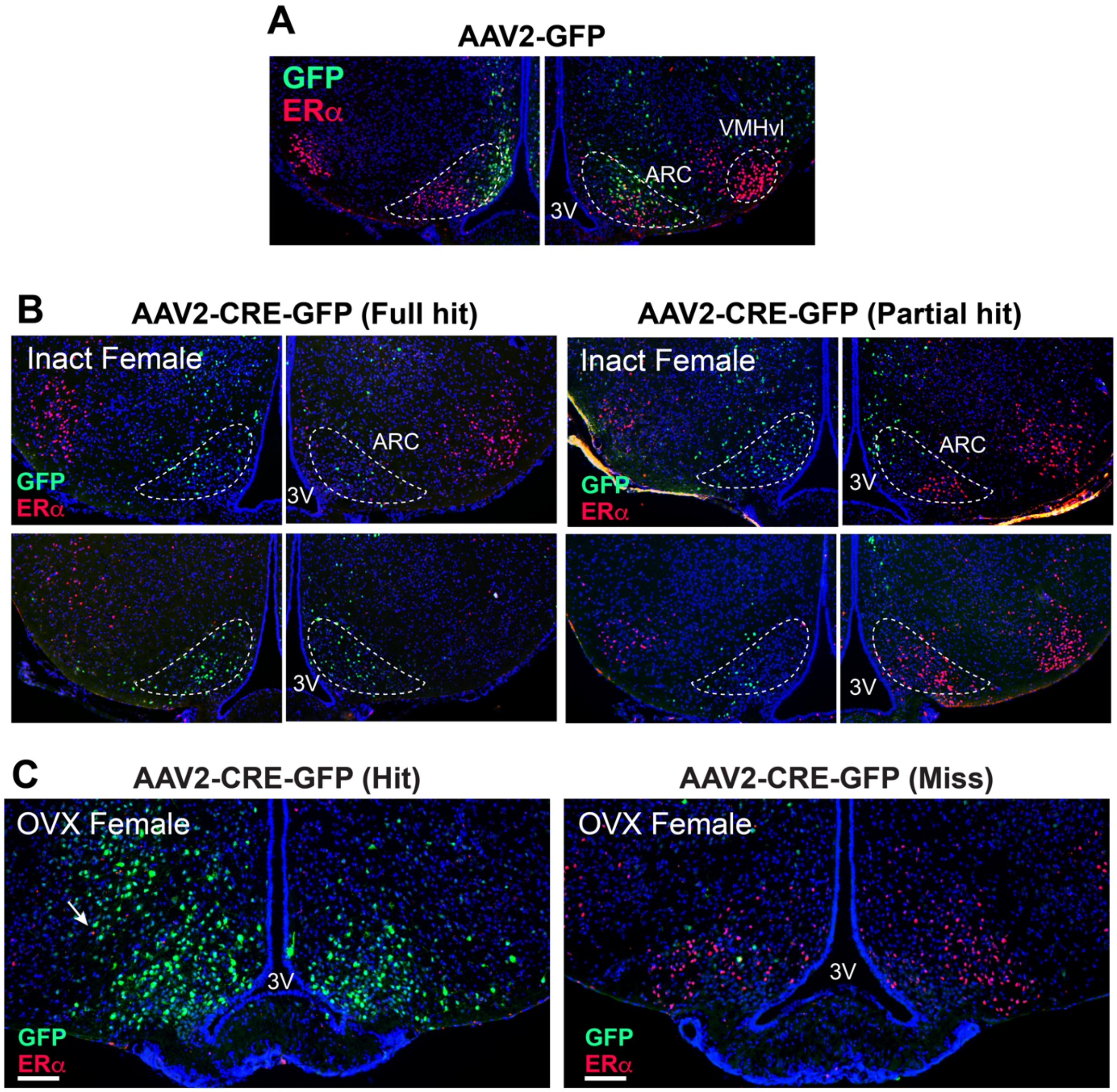

Supplementary Figure 4. Confirmation of ARC specific ablation of ERα in intact and OVX ERαKO^{ARC} females.

Representative images of ERα (red) and GFP (green) immunoreactivity shown for coronal brain sections (20 µm) to assess the extent of ERα ablation in the ARC (**A**) control (AAV2-GFP) or (**B**) AAV2-Cre-GFP (ERαKO^{ARC}) of intact females or from (**C**) estrogen-depleted OVX females. Representative images are shown for a Control, Full or Partial Hits in intact females (28 wks) and for a Hit or Miss in OVX females (~38 wks). Scale bar = 100 µm.

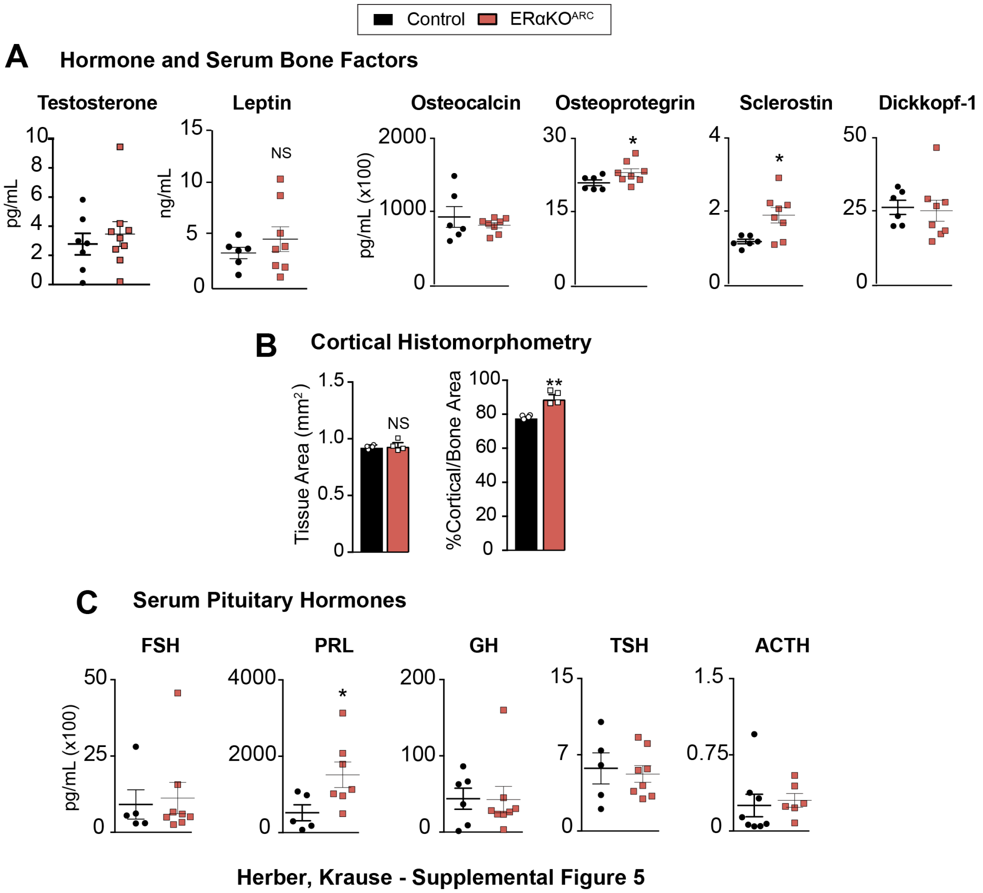

Supplementary Figure 5. Cortical bone area and pituitary serum hormones in ERαKO^{ARC} females.

(**A**) LC-MS/MS measurement of testosterone and ELISA quantification of circulating leptin and serum bone factors for control and ERαKO^{ARC} females 12 wks post viral infection (~28 wks). AAV2-GFP-ARC (black squares) and AAV2-Cre-ARC (red squares). (**B**) Morphometric analysis of cortical bone at the tibio-fibular joint in controls (n = 4, black bars) and ERαKO^{ARC} (n = 4, red bars) females. (**C**) Levels of circulating pituitary hormones (ρg/ml x 100) comparing control and ERαKO^{ARC} females 12 wks post viral infection. Error bars are ± SEM. Statistical analyses performed by Student's *t*-test, for Panels A, C, *p < 0.05; **p < 0.01.

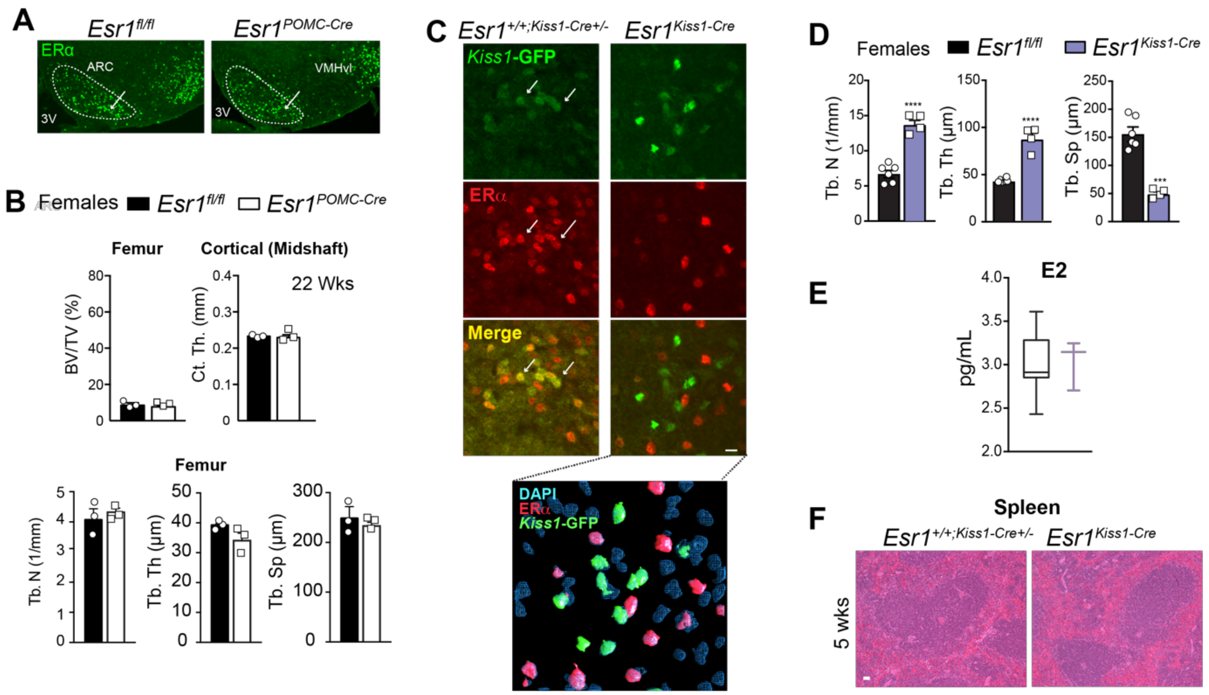

Herber, Krause - Supplemental Figure 6

Supplementary Figure 6. Increased bone mass in *Esr1*$^{Kiss1\text{-}Cre}$ but not *Esr1*$^{Pomc\text{-}Cre}$ female mice.

(**A**) Representative images of ERα (green) immunoreactivity shown for 20 μm coronal brain sections for *Esr1*$^{fl/fl}$ and *Esr1*$^{Pomc\text{-}Cre}$. (**B**) μCT quantitative morphometric indices for distal femur (n = 3 controls (black bars) and 3 mutants (open bars)) at 22 wks with legend above bar graphs. (**C**) Representative images of ERα (green) immunoreactivity shown for coronal brain sections for *Esr1*$^{fl/fl}$ and *Esr1*$^{Kiss1\text{-}Cre}$. Representative fields of ERα (red), GFP (green) and merged (Merge) images from ARC of *Esr1*$^{+/+}$*;Kiss1-Cre-GFP*$^{+/-}$ controls and *Esr1*$^{fl/fl}$*; Kiss1-Cre-GFP*$^{+/-}$ (*Esr1*$^{Kiss1\text{-}Cre}$) mutant females. Scale bar = 10 μm. Magnified surface map demonstrating no overlap of nuclear ERα and GFP staining in the ARC of *Esr1*$^{Kiss1\text{-}Cre}$ mutant females. (**D**) morphometric indices of distal femur (Esr1fl/fl, black bars and *Esr1*$^{Kiss1\text{-}Cre}$ purple bars) and (**E**) E2 levels (n =11 controls and 4 mutants) (center line = median; bounds are minimum to maximum). (**F**) Representative images of (20x) H&E stained spleens from control

and mutant females at 5 wks. Scale bar = 100μm. Error bars are ± SEM. Statistical analyses performed by Student's *t*-test for Panel D, ***p < 0.001, ****p < 0.0001.

Supplementary Table 1. Gene Expression In Femoral Bone And Bone Marrow.

GENE	Femur (Mean ± SEM)		Bone Marrow (Mean ± SEM)	
	Esr1$^{fl/fl}$	***Esr1***$^{Nkx2\text{-}1Cre}$	***Esr1***$^{fl/fl}$	***Esr1***$^{Nkx2\text{-}1Cre}$
Adbr2	1.1 ± 0.41	0.74 ± 0.17	2 ± 0.74	0.97 ± 0.1
Col2a1	0.77 ± 0.16	3.4 ± 1	0.85 ± 0.28	0.3 ± 0.08
Lepr	0.99 ± 0.08	1.6 ± 0.21	1.1 ± 0.16	0.32 ± 0.07*
Runx2	0.93 ± 0.07	1.5 ± 0.36	1.0 ± 0.18	2.6 ± 0.62*
Sp7	1.2 ± 0.30	5.7 ± 1.5*	1.1 ± 0.24	5.2 ± 3.8
Tcf7	0.77 ± 0.13	1.3 ± 0.48	0.62 ± 0.31	0.49 ± 0.15
Esr1	1.0 ± 0.10	0.96 ± 0.14		

Statistical analyses performed by Student's *t*-test, * = $p < 0.05$.

Supplementary Table 2. PCR Primers for Genotyping.

Allele	Forward	Reverse
Esr1 $^{fl/fl}$	TGGGTTGCCCGATAACAATAA	AAGAGATGTAGGGCGGGAAAA
Gt(ROSA)26Sor(CAG-tdTomato)	**WT allele**<br>AAGGGAGCTGCAGTGGAGTA	**WT allele**<br>CCGAAAATCTGTGGGAAGTC<br>**tdT Reporter**<br>GGCATTAAAGCAGCGTATCC
Nkx2-1Cre	CCACAGGCACCCCACAAAAAT	GCCTGGCGATCCCTGAACAT
Pomc-Cre	**WT allele**<br>TGGCTCAATGTCCTTCCTGG<br>**Cre Allele**<br>GAGATATCTTTAACCCTGATC	CACATAAGCTGCATCGTTAAG
Kiss1-Cre	GACCTAGGCTCTGGTGAAGTA	**WT allele**<br>AGCCTCCAGTGCTCACAGCAG<br>**Cre allele**<br>CTTGCGAACCTCATCACTCGTTGC
Dat-Cre	TGGCTGTTGGTGTAAAGTGG	**WT allele**<br>GGACAGGGACATGGTTGACT<br>**Cre Allele**<br>CCAAAAGACGGCAATATGGT
TH-Cre	GAG ACA GAA CTC GGG ACC AC	AGG CAA ATT TTG GTG TAC GG

Supplementary Table 3. Primer Pairs and Probes for BAT and Bone Markers.

RT-qPCR Primers		
**Gene**	**Forward Primer**	**Reverse Primer**
Ucp1	CACGGGGACCTACAATGCTT	TAGGGGTCGTCCCTTTCCAA
Pgc1α	CAGTACAGCCCCGATGACTC	GAAAGCTCGTCCACGTCAGAC
Pparγ	CGGGCTGAGAAGTCACGTT	TGTGTCAACCATGGTAATTTCAG
Prdm16	GAAGTCACAGGAGGACACGG	TCATTGCATATGCCTCCGGG
AdBr3	GGAAGCTTGCTTGATCCCCA	GCCGTTGCTTGTCTTTCTGG
Esr1	GAACGAGCCCAGCGCCTACG	TCTCGGCCATTCTGGCGTCG
RankL	TATAGAATCCTGAGACTCCATGAAAAC	CCCTGAAAGGCTTGTTTCATCC
Kiss1	TGCTGCTTCTCCTCTGT	ACCGCGATTCCTTTTCC
Pdyn	AGCTTGCCTCCTCGTGATG	GGCACTCCAGGGAGCAAAT
mCyclo	TGGAGAGCAGCACCAAGACAGACA	TGCCGGAGTCGACAATGAT
36b4	GGCACCGAGGCAACAGTT	TCATCCAGCAGGTGTTTGACA
Runx2	CGCACCGACAGTCCCAACTTCCTG	CACGGGCAGGGTCTTGTTG
Sp7	AGTGGGAACAAGAGTGAGCTG	TAGTGAGCTTCTTCCTGGGT
Wnt10b	CTGCGGATGGAAGGGTAG	GGGACTGAGCCAGGAACA
Sost	TAGCCCCGTGCCTCATCT	GGGATGGTGGGGAGGTCT
Bglap	GACTCCGGCGCTACCTTGGGTAAG	CCCAGCACAACTCCTCCCTA
Slc6a3	CTGATTGCCTTCTCCAGTTACA	GAAGCTCGTCAGGGAGGTAATG
