# Supplementary material for: Estrogen signaling in arcuate Kiss1 neurons suppresses a sex-dependent female circuit promoting dense strong bones
Source: Nat Commun. 2019 Jan 11;10:163. doi: 10.1038/s41467-018-08046-4 (PMC6329772; doi:10.1038/s41467-018-08046-4)
Supplement: Supplementary file 4 — Description of Additional Supplementary Files [file 41467_2018_8046_MOESM4_ESM.pdf]

Description of Additional Supplementary Files

File Name: Supplementary Data File 1
Description: DEGs in bone marrow of Esr1Nkx2-1Cre females

File Name: Supplementary Data File 2
Description: DEGs in microdissected ARC of Esr1Nkx2-1Cre females.
